# Simulation-Based Estimation of the Number of Cameras Required for 3D Reconstruction in a Narrow-Baseline Multi-Camera Setup

**DOI:** 10.3390/jimaging7050087

**Published:** 2021-05-13

**Authors:** Andreas Wachter, Jan Kost, Werner Nahm

**Affiliations:** Institute of Biomedical Engineering, Karlsruhe Institute of Technology (KIT), 76131 Karlsruhe, Germany; Jan.Kost@student.kit.edu (J.K.); Werner.Nahm@kit.edu (W.N.)

**Keywords:** surgical, microscope, surgical microscope, digital visualization, visualization system, common main objective, multi-camera setup, in silico, narrow baseline, 3D reconstruction, digital twin, simulation, surgical site model, MATLAB

## Abstract

Graphical visualization systems are a common clinical tool for displaying digital images and three-dimensional volumetric data. These systems provide a broad spectrum of information to support physicians in their clinical routine. For example, the field of radiology enjoys unrestricted options for interaction with the data, since information is pre-recorded and available entirely in digital form. However, some fields, such as microsurgery, do not benefit from this yet. Microscopes, endoscopes, and laparoscopes show the surgical site as it is. To allow free data manipulation and information fusion, 3D digitization of surgical sites is required. We aimed to find the number of cameras needed to add this functionality to surgical microscopes. For this, we performed in silico simulations of the 3D reconstruction of representative models of microsurgical sites with different numbers of cameras in narrow-baseline setups. Our results show that eight independent camera views are preferable, while at least four are necessary for a digital surgical site. In most cases, eight cameras allow the reconstruction of over 99% of the visible part. With four cameras, still over 95% can be achieved. This answers one of the key questions for the development of a prototype microscope. In future, such a system can provide functionality which is unattainable today.

## 1. Introduction

Since the late 1980s, visualization systems have received increasing attention in medicine [1]. Medical visualization systems are used to graphically display image data or three-dimensional (3D) volume data. These systems provide a broad spectrum of information to support the diagnostic process, therapy (e.g., surgical interventions) and post-treatment care. In recent years, the increasing availability of digital pre- and intra-operative medical data has made 3D visualization and information fusion more desirable. Previously, the sole focus lay on the visualization of the surgical site. However, now the interaction of observers with the data is gaining importance. If the information is available entirely in digital form, the user can view it on a monitor and manipulate it freely, e.g., in rotation, scale, brightness and contrast. This will aid in the understanding of complex anatomy, e.g., in the field of radiology [2]. Such visualization systems can even be extended into augmented or virtual reality (AR or VR). In many use cases, the acquisition and analysis of the data are decoupled. Since the pre-recorded 3D data is fused and then viewed offline, there is no limit to the number of observers. Observers in this context are to bee understood as, e.g., medical staff or staff in medical training who view the previously recorded 3D data. These observers’ views are flexible and independent of each other. The field of surgery is not afforded this luxury yet. The surgical site itself is viewed through a microscope, endoscope, or laparoscope, with no digital representation available. This precludes both the aforementioned information fusion and freedom of manipulation, leaving several aspects to be desired in contemporary surgical visualization systems. While endoscopy, laparoscopy, and microsurgery share similar limitations, we will take a closer look at the shortcomings of surgical microscopes here, since these devices are the focus of our work. In principle, however, the approach we will propose to mitigate said limitations is also applicable to endoscopy and laparoscopy. Ergonomics pose a problem in surgical microscopes since surgeons must remain in unfavorable positions for a long time during certain procedures, causing discomfort and muscle pain [3,4]. Furthermore, spatial and optical limitations allow only two simultaneous observers. Further observers require added opto-mechanical components, increasing cost, complexity, weight, and bulk of the microscope head. The added weight, in turn, complicates the construction of the balancing systems required for easy handling of the microscope by the surgeon [5]. In order to increase the overall number of observers and enhance the freedom of movement, some manufacturers employ a digital 3D streaming approach in their newest surgical microscopes. Examples for this can be found in the ZEISS KINEVO^®^ [6] or *Munich Surgical Imaging*’s ARRISCOPE [7]. Here, users observe the microscope image on a monitor [8,9] or a head-mounted display [9,10,11], which alleviates the previously mentioned ergonomic problems. A further advantage of streaming systems is the inclusion of remote observers, e.g., for training purposes [12]. Especially with 3D streaming, the additional depth perception leads to a better understanding of the anatomical structures and the surgical area [13].

Despite the improvements this feature provides, it does not solve the fundamental problem of dependent co-observers. The views of the co-observers are usually linked to the main observer [14]. This makes the hand-eye coordination required for high-precision work very challenging for the assistants. They must mentally rotate and translate the scene in relation to their point of view, which makes it difficult for untrained assistants to collaborate with the surgeon, further increasing their mental workload during the surgical procedure. This can adversely affect surgical performance [15].

An optimal solution for multiple co-observers (assistants) with views independent of the surgeon is required.

As mentioned above, this problem has already been solved in fields, such as radiology, where the data is completely digital and available in 3D. Therefore, the question arises, whether the same can be achieved in a surgical microscope.

The surgical site would have to be digitized, creating a 3D digital twin. Several digitization techniques are available for this purpose including scanning systems, based on time-of-flight approaches, and photogrammetric multi-camera systems. Time-of-flight systems only yield structural information and do not provide textural information, which is particularly important in medical applications. Photogrammetric approaches are preferable since they provide textural information. This advantage comes at the cost of more challenging extraction of accurate structural information. Increasing the number of independent camera views used for reconstruction can help mitigate this drawback. For cost and space reduction, as well as for increased usability and efficient data handling, the number of cameras should, however, be minimized. This raises the question: How can we photogrammetrically reconstruct a 3D digital twin of a surgical site for maximum observational flexibility and unlimited co-observers while keeping the number of cameras minimal? While there are many conceivable approaches to the solution of this problem, we chose to find the theoretical optimum under the constraints of a common main objective (CMO) surgical microscope. Microscopes with this proven optical setup are very common. Most importantly, adding a 3D digital visualization system as envisioned above to such a microscope would allow keeping the existing analog observation system as a fallback. Thus, if the main visualization system were to fail during an operation, the surgeon would still be able to continue the procedure using the oculars. Additionally, surgeons worldwide are experienced in the use of current surgical microscopes. Therefore, adding a visualization method to the existing type of microscope would aid acceptance of the new systems and smooth the transition from existing devices.

By choosing this type of microscope, we subject ourselves to the limitation of the available baseline for stereo camera pairs used for reconstruction. CMO microscopes must be considered as narrow baseline systems since their inter-camera-angle (stereo angle) α is typically less than $10^{\circ }$ [16]. While the benefits of using true multi-view camera setups for 3D reconstruction of surfaces have been investigated in previous work, this has only been done for wide-baseline camera systems with $15^{\circ }<\alpha <45^{\circ }$:

Rumpler et al. [17] evaluated the influence of the number of cameras on depth accuracy and robustness of the 3D reconstruction of an urban scenery using multi-view camera setups. The authors arranged cameras in a 2D grid, spaced such that 80% of the image content overlapped. They found that the 3D reconstruction accuracy increases with the individual number of available measurements (i.e., camera views). Sing Bing et al. [18] further showed that using more than two camera images leads to an increase in the quality of reconstruction.

To the best of our knowledge, no such study has yet been performed for a narrow baseline system like a surgical microscope.

**We therefore seek to answer the following question:** What is the minimal number of independent camera views required to create an adequately complete digital twin of a microsurgical site, using a narrow baseline CMO camera setup?
**Our hypothesis is:** At a finite number of cameras, the 3D reconstructable area of the digital twin reaches a point where a further increase in camera views yields only a negligible increase in the reconstructable area.

It is necessary to define what should be considered adequately complete in this context. A 3D visualization system in a surgical microscope should not hide anything that is already visible through the existing observation channels. Therefore, we define our ground truth for a truly complete digital twin of a surgical site as: everything a surgeon could see at any point during a complete fly-around of the site while looking through the main observation channel of a CMO surgical microscope. In other words, a surgical site reconstruction would be considered complete if it allows the rendering of any view that is possible with the analog microscope from a given microscope head pose—without any areas missing in the rendered images.

Asking for merely adequate completeness, we can somewhat relax this requirement. A reconstruction is considered adequately complete if it lacks only small regions of the ground truth and if these are located exclusively in boundary regions underneath occluding structures. Boundaries in this context are the borders between reconstructable and non-reconstructable regions of the ground truth.

We had no access to a testbed for different physical camera constellations in a CMO setup. Three-dimensional surgical site models and image series that we could have used to calculate such models were likewise unavailable to us. We therefore created in silico models of surgical sites, as well as the surgical microscope camera system itself. We present these models in this contribution. Using our models, we performed simulated 3D reconstructions of sites to determine the performance of different narrow-baseline multi-camera setups. Based on these experiments, we estimate optimal camera numbers for the reconstruction of digital twins of typical surgical sites. Finally, we also present the MATLAB [19] simulation framework, which we developed for our experiments. The framework code are available under [20].

## 2. Methods

### 2.1. Mesh Design

Meaningful comparison of the performance of different simulated camera setups with respect to the 3D reconstruction of 3D surfaces requires surface models representative of the use-case. To this end, we created models that realistically depict challenging neurosurgical sites with deep channels and the anatomy therein. The underlying simulation framework requires the models to be manifold 3D triangle meshes in the binary STL format.

**Figure 1:**
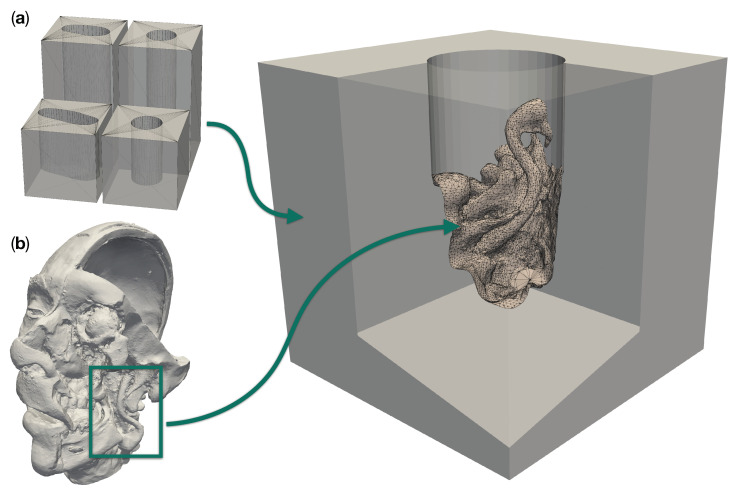
Design components for a realistic site models with a patient anatomy in a deep operative corridors. (**a**) shows the four different operative corridors used: Two cylindrical channels with a depth of 50 mm and 100 mm, respectively, as well as two channels of the same depths with a deformed elliptical cross-section. (**b**) Three-dimensional model of a plastinated and dissected human head. The box highlights the region which was merged into an empty cylindrical channel to create the particular model to the right.

We modeled the operative corridors as channels with circular and deformed elliptical cross sections [21,22], as shown in Figure 1a. The upper surface of the channel block, which contains the channel opening, will be referred to as “top surface” in the following. This surface is to be oriented towards the cameras. A diameter of 25 mm was chosen for the cylindrical channel, while the elliptical cylinder was made approximately 42.5 mm long and 22.5 mm wide. We used two different depths for each corridor type: 50 mm and 100 mm. The diameters and depths were selected based on the data in Table 1, as well as the typical lengths of microsurgical instruments, ranging from 8.5 cm to 11 cm [23]. The surgical site anatomy inserted into the corridor models was taken from a plastinated and dissected patient head model (shown in Figure 1b), which was kindly provided to us by the University of Hawaii [24]. We specifically used regions directly posterior to the mandible on both sides of the head. These regions include segments of the internal and external carotid arteries.

**Figure 2:**
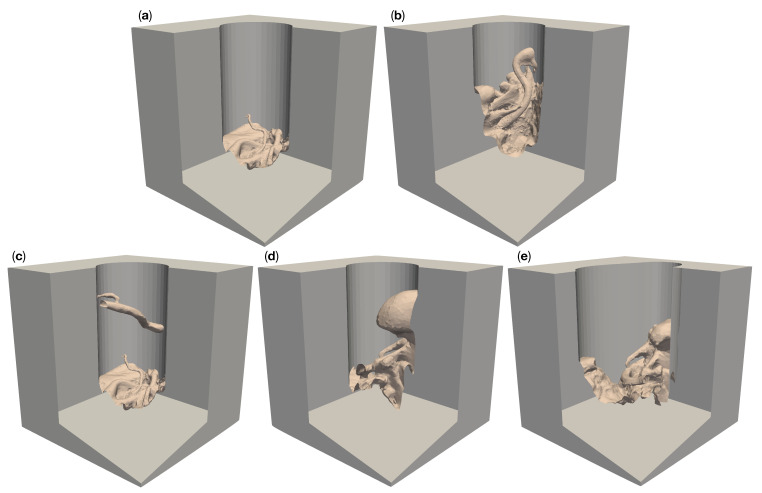
Five different artificial patient surgical site models with a 50 mm deep operative corridors. “Circ flat anatomy”, shown in (**a**), contains a rather flat topology. “Circ angled anatomy” in (**b**) features a network of arteries, tissue furrows and undercuts, which are set at an angle in the corridor. “Circ artery” (**c**) has the same flat topology as in (**a**) at the bottom of the operative corridor, but adds an artery spanning the operative corridor. (**d**) shows a topology consisting of a mixture of arteries, tissue furrows, undercuts, dominated by a large overhang protruding from the channel wall. This model is called “circ overhang”. The model in (**e**) combines features from (**b**,**d**), set in an operating corridor with deformed elliptical cross-section, hence its name: “elliptical channel”.

**Figure 3:**
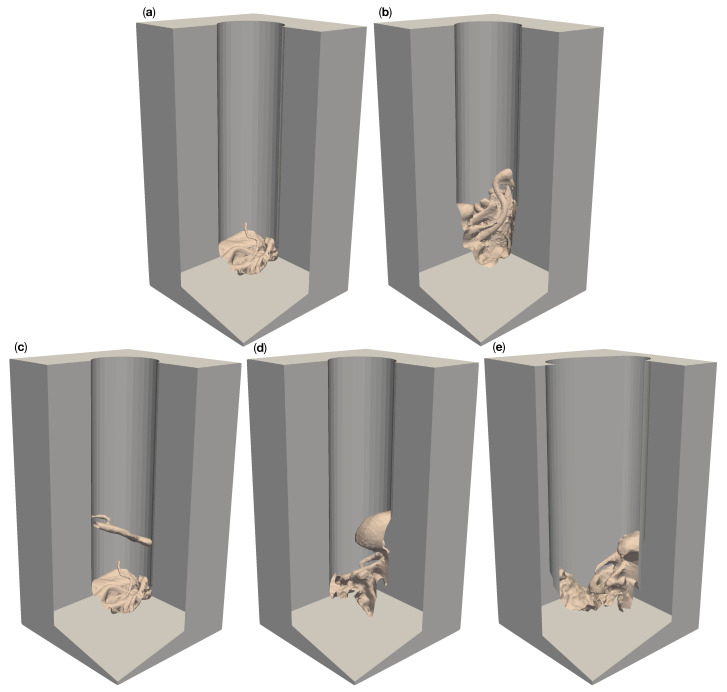
Five different artificial patient surgical site models with 100 mm deep operative corridors. “Circ flat anatomy”, shown in (**a**), contains a rather flat topology. “Circ angled anatomy” in (**b**) features a network of arteries, tissue furrows and undercuts, which are set at an angle in the corridor. “Circ artery” (**c**) has the same flat topology as in (**a**) at the bottom of the operative corridor but adds an artery spanning the operative corridor. (**d**) shows a topology consisting of a mixture of arteries, tissue furrows, undercuts, dominated by a large overhang protruding from the channel wall. This model is called “circ overhang”. The model in (**e**) combines features from (**b**,**d**), set in an operating corridor with a deformed elliptical cross-section, hence its name: “elliptical channel”.

We created a total of ten different reference models with five different complex surgical site topologies, each embedded into two different corridors of the depths discussed above. The resulting models cover anatomic structures of varying complexity. The model “circ flat anatomy” in Figure 2a and Figure 3a contains a rather simple flat topology with only small blood vessels protruding close to the bottom. Adding a large diameter vessel spanning the channel, approximately 3 cm above the existing anatomy, yields the model “circ artery”, shown in Figure 2c and Figure 3c. Figure 2b and Figure 3b shows “circ angled anatomy”. This model contains a different topology consisting of a network of arteries, tissue furrows and undercuts, set at an angle in the operative corridor. A similar topology to this, albeit overshadowed by a large bulging structure protruding into the channel, is used in “circ overhang”, as shown in Figure 2d and Figure 3d. The final model, “elliptical channel”, in Figure 2e and Figure 3e, combines features from the aforementioned models (such as overhangs, tissue furrows, and undercuts) in the deformed elliptical channel.

#### Mesh Creation

The models presented above were created using Autodesk Inventor^®^ 2020 and Autodesk Meshmixer^®^, as well as Blender^™^. Inventor^®^ was used to create the channel blocks, while Meshmixer^®^ served to cut and edit regions of anatomy from the head model shown in Figure 1b. The channel blocks and cut-out anatomy were merged in Blender^™^ and exported as binary STL from there.

### 2.2. Camera Setup

Our simulated camera setup takes into account the restrictions imposed by the CMO optical setup commonly used in surgical microscopes. We chose a camera arrangement that reflects these restrictions in the manner described in the following paragraph. Each individual camera model consists of two separate components: the positioning of the cameras and the simulated image acquisition from each camera. The cameras in our work were modeled as pinhole cameras with restricted field of view (FOV). These camera models do not include rasterized image generation. Since the resulting images are in continuous space, we do not explicitly model any 3D reconstruction algorithm. We assume rather that any point is “reconstructable” in 3D if it is visible to at least T cameras.

We also assume homogeneous and gapless illumination of every point on the surgical site model, thus disregarding any influences of the lighting. As a result, only the positioning of the cameras and their viewing direction influence the visibility—and by extension, the reconstructability—of a point on an object in space.

#### 2.2.1. Surgical Microscope Model

In order to keep our setup model simple, we decided not to explicitly model any optical components. Therefore, we treat all lenses in our models as ideal. In addition to this, the beam paths in the portion of the optical system inside a CMO microscope (i.e., between the CMO lens and the cameras) are parallel. These two conditions—combined with the assumption that the zoom system is set to a magnification of 1—allow us to neglect the aforementioned portion of the optical system in our model setup. We can, therefore, move the cameras forward onto the principal plane of the CMO lens.

We can then further omit the CMO lens itself from the setup model. To retain the equivalence of the model with the real optical system after this, we must align each camera so that the respective principal ray becomes collinear with its previously refracted counterpart. After this, all cameras in the setup are confocal in the sense that the principal rays intersect in the central reference point $\vec{F}$. Consideration must also be given to the magnification. After omission of the CMO lens, magnification depends on the distance of a camera from $\vec{F}$. We assume that all cameras in the setup are identical and the depth of field of the cameras is unlimited. If the images of an object located at $\vec{F}$ are to have uniform magnification in all cameras, these cameras must be placed on a sphere centered on $\vec{F}$, thus maintaining equidistance from the object.

**Figure 4:**
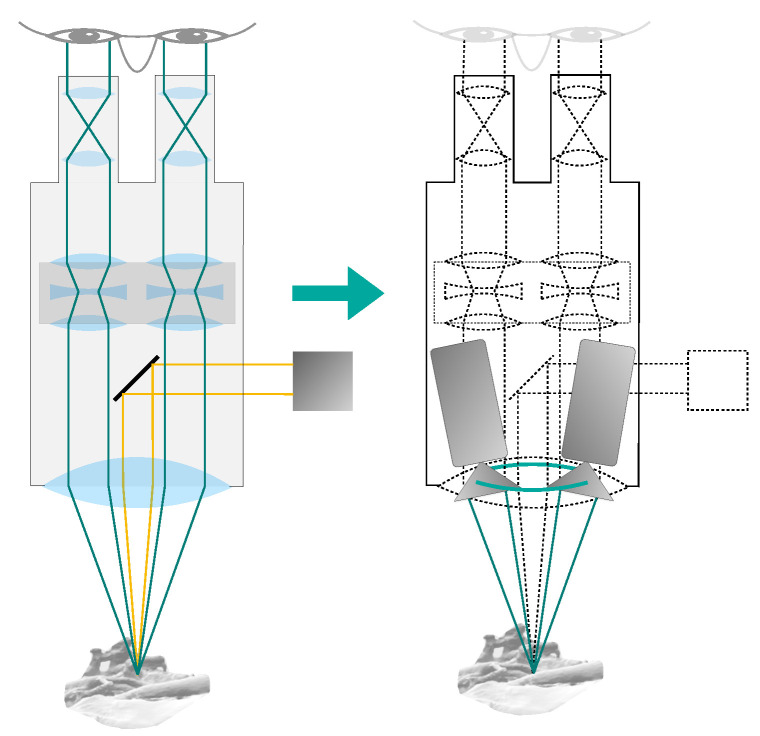
Simplifications used in our surgical microscope model. The optical setup within the microscope is omitted and the main objective lens is assumed to be ideal. The cameras are placed directly on the non-refracted ray paths, at a distance from the object equal to the focal length of the main objective lens. The cameras are located on a ring with the diameter of the CMO lens (shown in green).

In a stereo camera setup, it is inherently better to use a large baseline, since an increased baseline leads to better accuracy of reconstruction via triangulation and, at the same time, generally allows the system to reconstruct more of the surface area. This is especially true for objects that extend perpendicularly to the cameras’ viewing directions. We, therefore, chose to arrange the cameras in our models on a ring with a diameter equivalent to the maximum usable baseline $b_{main}$ of real-world CMO surgical microscopes. This is limited by the CMO lens and typically ranges from 20 mm to 30 mm [25]. The arrangement on such a circle ensures that any two cameras, which lie exactly opposite each other, constitute a stereo pair with the greatest realistically feasible baseline. Figure 4 illustrates the aforementioned simplification steps and considerations.

**Figure 5:**
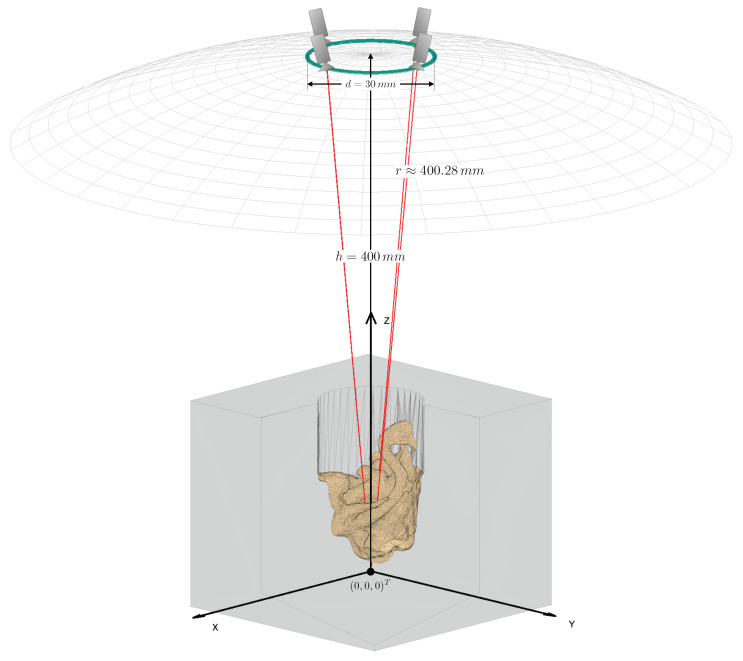
Schematic of the final camera setup model with an example mesh and four cameras. The mesh is centered at the origin of the world coordinate system. The cameras are placed on a ring (green) above the mesh. The wireframe hemisphere lies at a distance from the origin equal to the common main objective lens’ focal length. It marks the surface on which cameras may be positioned. The optical axes of the cameras are shown in red. The cameras all “look at” the central reference point, which is the origin in this case. The figure is not to scale in the z-direction. It has been shortened for illustration purposes.

Our final camera setup model is shown in Figure 5. The setup allows an arbitrary number of cameras to be arranged on a ring with a diameter $d=b_{main}$. This ring is situated at a distance *h* in the z-direction from a main reference point $\vec{F}$, where all cameras in the setup focus on. In the simulated microscope, *h* is equal to the microscope’s working distance $L_{wd}$. These mentioned parameters can be chosen by the user and fed into our simulation.

#### 2.2.2. Camera Model

We modeled each individual camera as a pinhole camera according to Morvan [26]. The pinhole model consists of two components: An extrinsic matrix $M_{ext}\in R^{3\times 4}$ that contains the camera’s position and orientation, and an intrinsic matrix $M_{int}\in R^{3\times 3}$, which defines the projection onto its image plane. The calculation of the camera matrices is described in the following, beginning with the extrinsic matrix. The camera poses were specified using the *Look-At* parametrization known from the *openGL* rendering-API [27]. 

The look-at camera pose is defined by three components:

- The position of the camera’s aperture: $\vec{a}\in R^{3}$.
- The direction $\vec{l}\in R^{3}$ in which the camera looks.
- The direction that is to be considered “up” in the camera coordinate system: $\vec{u}\in R^{3}$.

**Figure 6:**
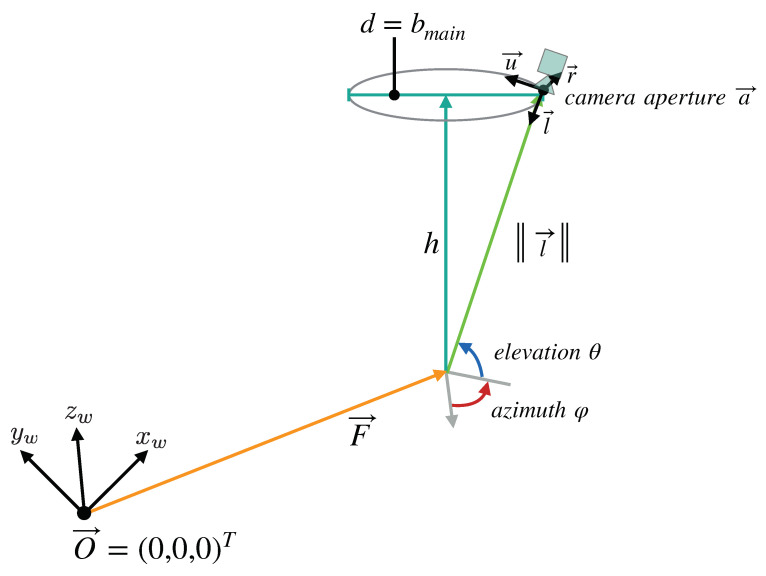
Overview of the parameters that define the camera setup model we devised for our simulation. The cameras lie on a ring with the diameter *d*, at the distance *h* from the reference point $\vec{F}$, which all cameras look at. The distance *h* is equivalent to the working distance of the fully digital microscope simulated by the model, while *d* is the maximum baseline $b_{main}$ allowed by the microscope’s main objective. The points on the ring can be described in spherical coordinates relative to $\vec{F}$. In these coordinates, $∥\vec{l}∥$ is equal to the main objective’s focal length.

The camera coordinate system axis directions are derived from the three components of the pose: The forward (look-at) axis in our case $\vec{l}=\frac{\vec{a}-\vec{F}}{∥\vec{a}-\vec{F}∥}\in R^{3}$ is the normalized vector from the aperture to the central reference point $\vec{F}$. The right-axis $\vec{r}=\frac{\vec{u}\times \vec{l}}{∥\vec{u}\times \vec{l}∥}\in R^{3}$ is the normal of the cross product of the up direction with the look-at axis direction. And the up-axis $\vec{u}=\vec{l}\times \vec{r}\in R^{3}$ is the cross product of the look-at axis direction with the right-axis direction. An overview of the different directions and axes is given in Figure 6.

The camera’s extrinsic matrix is composed of the 3 × 3 rotation matrix component R and the 1 × 3 translation vector t. The camera coordinate system’s axes define R as follows:

(1) $$R=\left(\begin{array}{ccc} r_{1} & r_{2} & r_{3}\\ u_{1} & u_{2} & u_{3}\\ -l_{1} & -l_{2} & -l_{3} \end{array}\right)$$

The translation vector t is the product of R with the aperture position $\vec{a}$:

(2) $$t=-R\;\vec{a}$$

Using R and t, the extrinsic matrix can be constructed as:

(3) $$M_{ext}=\left(\begin{array}{cccc} r_{1} & r_{2} & r_{3} & t_{1}\\ u_{1} & u_{2} & u_{3} & t_{2}\\ -l_{1} & -l_{2} & -l_{3} & t_{3} \end{array}\right)$$

While the extrinsic matrix depends on the individual cameras’ pose, the intrinsic matrix $M_{int}$ is universal to all cameras in our simulation:

(4) $$M_{int}=\left(\begin{array}{ccc} -\frac{f}{s_{x}} & s_{s} & o_{x}\\ 0 & -\frac{f}{s_{y}} & o_{y}\\ 0 & 0 & 1 \end{array}\right)$$

Here, the image distance *f* in the camera is the distance from the pinhole to the image plane (i.e., the sensor). In conjunction with the pixel sizes in the x and y direction $\left(s_{x},s_{y}\right)$, this determines the scaling from world coordinates (in length units) to image coordinates (in pixels). The sensor skew coefficient $s_{s}$ is not relevant to our experiments, since we are not rendering the cameras images. The simulation framework therefore assumes rectangular sensors in all cases $\left(s_{s}=0\right)$. The principal point offset $\left(o_{x},o_{y}\right)$ describes the shift of the camera sensor w.r.t. the camera’s principal axis. Like the skew coefficient, this is not relevant to our application. In the framework $\left(o_{x},o_{y}\right)$ is always (0,0).

The simulation allows the user to specify sensor size (in length units) and resolution (in pixels). The sensor size and image distance together determine the field of view (FOV) of the cameras. The sensor resolution only affects image space coordinate axis scaling via the pixel sizes. It has no influence on the results of the simulated reconstruction since no rasterized images are produced anywhere in the program sequence.

For the purposes of our simulation, the projection matrix $P=M_{int}\;\cdot \;M_{ext}$ of each individual camera fully describes the projection of a point, given by its homogeneous coordinates ${\left(x,y,z,1\right)}^{T}$, from world space to this camera’s image coordinate system.

### 2.3. Reconstruction Algorithm

**Figure 7:**
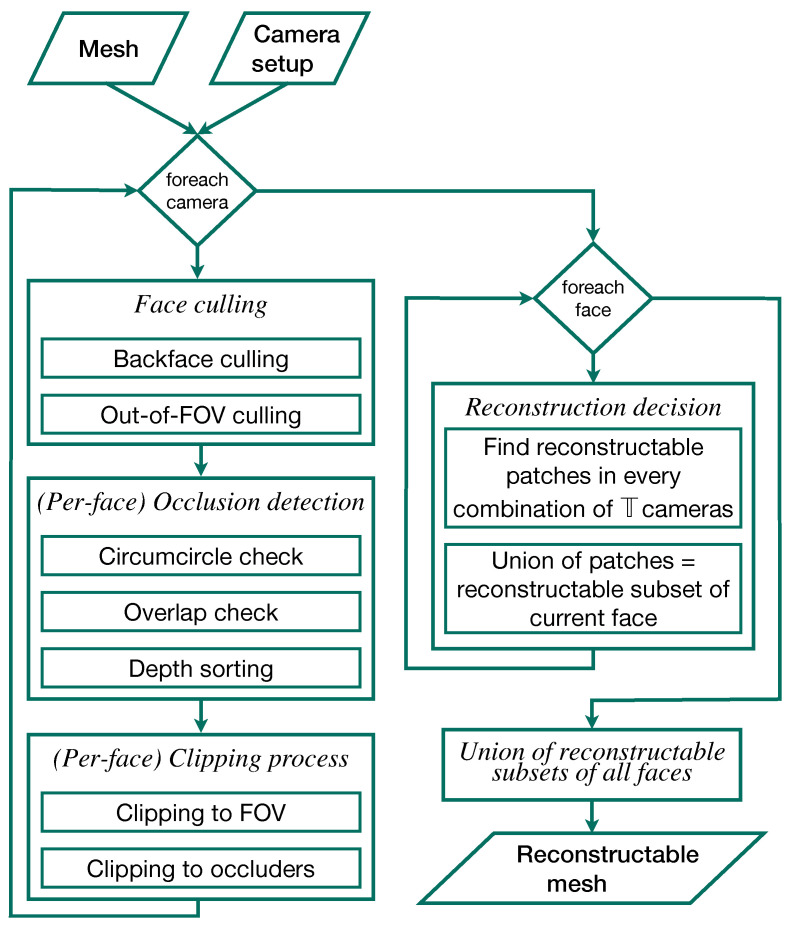
Overview flow chart of the individual steps of the 3D reconstruction simulation. The camera setup and surgical site models comprise the *input*. The *output* is the reconstructable subset of the input mesh in 3D.

Our simulation framework finds the reconstructable subset of each face in the input mesh. To this end, the program must first find the parts of each face that are visible to the individual cameras in the simulated setup. This first step must be done once for each camera and consists of several operations on all faces, performed in the following order: Invisibility culling, occluder detection, clipping to the camera’s FOV, if applicable, and, finally, clipping to a set of known occluders. From the resulting subsets of each face which are visible to each of the cameras, the simulation can then derive the reconstructable subset of the face in question. The union of these reconstructable subsets of the individual faces comprises the reconstructable subset of the input mesh. The individual steps of the algorithm are described in more detail below. A schematic overview of the complete program is given in Figure 7. For explanations of the low level methods used throughout the algorithm, please refer to Section 2.3.5.

#### 2.3.1. Face Culling

The algorithm first eliminates backfaces and faces on the contour of the input model, which are by definition invisible to the camera. The dot products of these faces’ normal vectors with the current camera’s look-at direction $\vec{l}$ are positive or zero. Only faces with normals oriented partially towards a camera’s aperture are considered candidates for visibility to this camera.

The possible candidates are then projected into image space. There, they are tested for location within or without the FOV. If a face’s image is fully outside the FOV rectangle, it is also culled. Faces whose image intersects the FOV borders are marked for clipping against the FOV at a later point. Faces that are fully inside the FOV are kept and not altered.

#### 2.3.2. Occlusion Detection

The faces that survived culling are then tested for mutual occlusion. This itself is a three-step process, also in image space, beginning with a circumcircle check. For this, a list of potential occlusion partners (occluders or occludees) is started for each surviving face. Every other surviving face, whose circumcircle overlaps with the subject face’s circumcircle, is entered into this list. If the circles do not overlap, the potential partner is not included in this list, since occlusion between the two faces can be definitively ruled out. Each face’s image is then tested against the image of each of its potential occlusion partners. If the two images overlap, occlusion is confirmed. In this case, the minimal depth of the original 3D faces is compared. The minimal depth in this context is the smallest distance in world space from the camera’s aperture to any of a face’s three vertices. The face with the greater minimal depth is occluded by the other face. The shallow face is added to the deeper face’s list of known occluders.

#### 2.3.3. Clipping Process

Having compiled the lists of known occluders, the hidden portions can be removed from each face in each camera’s view. First, a subject face is clipped against the current camera’s FOV if it has been marked for this in the culling step. Only the portion of the face’s image that is inside the FOV rectangle is kept.

After having been clipped to the FOV, the region of the subject face inside the FOV is then further clipped against its known occluders. If no occluders were previously found, this step is skipped. Otherwise, the images of all known occluders are merged into as few polygons as possible, often one, against which the remaining subject region is then clipped. The portion of the subject region outside of the merged occluder polygon is kept. This portion is the subset of the subject face that is visible to the current camera. This set may be empty. In this case, the subject face is marked as invisible to the current camera and ignored downstream in the algorithm.

#### 2.3.4. Finding the Reconstructable Subset of the Mesh

In our simulation framework, the user must specify a reconstructability threshold T. This is the minimal number of cameras deemed necessary for the 3D reconstruction of a given point on the mesh. A surface patch on the input mesh is not part of the reconstructable subset if it is visible to less than T cameras. Under ideal circumstances, T=2. Based on the given reconstructability threshold, the simulation finds the reconstructable subset of each individual face and combines these subsets of all faces into a new mesh comprising the reconstructable subset of the input mesh.

The simulation begins this by listing, for each individual face, the cameras that it is at least partially visible to. If the number of cameras in this list for a given face is less than T, the face is not reconstructable. Otherwise, the visible subsets of the face are recovered from each of the camera’s views. Then, all possible combinations of T camera views are determined. The intersection of the visible fragments from each of these combinations of camera views constitutes a region of the face that is reconstructable with the simulated camera setup. The union of all these intersections, i.e., the union of all regions of the face that are reconstructable with a combination of T cameras, comprises the entire reconstructable subset of the face in question. These binary operations on 3D polygons are done using the *polyshape*-based 2.5D method described in Section 2.3.5.

In the following, a formal description of the reconstructable subset of the input mesh is given:

Let the input mesh

(5) $$\begin{array}{cc} U_{N}:= & \left\{F_{1},\ldots ,F_{N}\right\} \end{array}$$

be the set comprising all *N* faces, and let

(6) $$\begin{array}{cc} V_{M}:= & \left\{c_{1},\ldots ,c_{M}\right\} \end{array}$$

be the set of all *M* simulated cameras. Then,

(7) $$\begin{array}{cc} \varphi_{i} & :V_{M}\mapsto W_{i},\;f_{i}^{j}=\varphi_{i}\left(c_{j}\right) \end{array}$$

is the portion of the face $F_{i}$ that is visible to the camera $c_{j}$. $W_{i}$ comprises all polygonal partial areas of $F_{i}$. The list $L_{i}$ of cameras that see at least a partial area of $F_{i}$ is:

(8) $$\begin{array}{cc} L_{i}:= & \left\{c_{j},j\in \left\{1,\ldots ,M\right\}|f_{i}^{j}\neq \emptyset \right\} \end{array}$$

We denote the set of combinations of T cameras from this set $C_{i}$, which we define as follows:

(9) Ci:=LiT=l1,…,lP,wherelk=ck1,…,ckT,k∈1,…,P

Therein, *P* is the cardinality of $C_{i}$:

(10) P=|Ci|=|Li|T

Using this notation, the reconstructable subset *R* of the input mesh is described as:

(11) $$\begin{array}{cc} R & =\overset{N}{\underset{i=1}{⋃}}\overset{{:=R}_{i}}{\overset{︷}{\overset{P}{\underset{k=1}{⋃}}\underset{{:=r}_{i_{k}}}{\underset{︸}{\overset{T}{\underset{t=1}{⋂}}\varphi_{i}\left(c_{k_{t}}\right)}}}},\;\mathrm{where}\;c_{k_{t}}\in l_{k},\;l_{k}\in C_{i} \end{array}$$

Here, $R_{i}$ denotes the reconstructable subset of the face $F_{i}$. This subset $R_{i}$ consists of the regions $r_{i_{k}}\in W_{i}$ which are reconstructable with each individual camera combination $l_{k}$.

#### 2.3.5. Occlusion Detection, Clipping, and Binary Operations on 3D Polygons

In the following, we outline the underlying methods used by the reconstruction algorithm in Section 2.3.

##### 2.5D Occlusion Detection

Finding out whether a given polygon in 3D overshadows a subject polygon in a given camera’s view might be approached by determining if the subject polygon lies partially or entirely within a volume in space, defined by the projection of the potential occluder polygon from the camera’s aperture into infinity. However, this is complex and expensive. MATLAB offers far superior built-in options for 2D polygons. Using MATLAB “*polyshape*” objects created with the vertices of the subject and occluder polygons in the current camera’s image space, the problem is reduced to calling *overlaps (subjectImage,occluderImage)*. The result translates immediately to 3D with no additional steps required.

##### 2.5D Clipping

Two different clipping scenarios can occur in our simulation. First, faces might be clipped to the camera’s FOV. Here, the FOV in image space is a rectangle, defining a *polyshape*. The image of the subject’s face is a triangle, defining the second *polyshape*. We are interested in the portion of the face that is inside the FOV. Therefore, the 2D clipping result is the region shared by both polyshapes: *intersect(FOVrectangle,subjectImage)*. To obtain the 3D-result, the vertices of the resulting polygon are back-projected into 3D. The correct 3D vertices are found by the intersection of the vertex rays with the plane spanned by the original 3D subject polygon.

Occlusion clipping follows the same principle as clipping to the FOV but uses *subtract(subjectImage,occluderImage)* to obtain the portion of the subject *polyshape* that is outside the occluder *polyshape*.

##### Binary Operations on 3D Polygons

In the last step of the reconstruction pipeline, the simulation framework must perform boolean operations on all visible subsets of a given face from the different camera views. Each of these subsets is a group of one or more polygonal fragments in 3D. In order to use *polyshape* methods on them, they must first be transformed into 2D. We chose to avoid dependence on camera parameters by not projecting the fragments into any of the camera’s image spaces. Instead, the fragments’ vertices are transformed into a local 2D coordinate system in the plane spanned by the parent face’s vertices $v_{1}$, $v_{2}$ and $v_{3}$. Having computed the transformation as shown here:

$$\begin{array}{cc} \vec{o} & ={\left(0,0,0\right)}^{T}\\ {\vec{o}}^{\prime } & \vec{v_{1}}\\ {\vec{x}}^{\prime } & =\frac{\vec{v_{2}}-{\vec{o}}^{\prime }}{∥\vec{v_{2}}-{\vec{o}}^{\prime }∥}\\ {\vec{y}}^{\prime } & =\frac{\vec{n}\times {\vec{x}}^{\prime }}{∥\vec{n}\times {\vec{x}}^{\prime }∥}\;\mathrm{where}\;\vec{n}={\vec{x}}^{\prime }\times \left(\vec{v_{3}}-{\vec{o}}^{\prime }\right), \end{array}$$

any 3D point $\vec{p}$ can be transformed into and from its local 2D representation ${\vec{p}}^{\prime }$ with the following operations:

(12) $$\begin{array}{cc} {\vec{p}}^{\prime } & =\left(\begin{array}{c} p_{1}^{\prime }\\ p_{2}^{\prime } \end{array}\right)=\left(\begin{array}{c} \left(\vec{p}-{\vec{o}}^{\prime }\right)\;\cdot \;{\vec{x}}^{\prime }\\ \left(\vec{p}-{\vec{o}}^{\prime }\right)\;\cdot \;{\vec{y}}^{\prime } \end{array}\right) \end{array}$$

(13) $$\begin{array}{cc} \vec{p} & ={\left(p_{1},p_{2},p_{3}\right)}^{T}={\vec{o}}^{\prime }+p_{1}^{\prime }\;\cdot \;{\vec{x}}^{\prime }+p_{2}^{\prime }\;\cdot \;{\vec{y}}^{\prime } \end{array}$$

After transforming each fragment’s vertices using Equation (12) and generating *polyshape* objects using the transformed vertices, the desired boolean operations can be performed conveniently. The results of the boolean operations can then be transformed back into 3D with Equation (13).

## 3. Results

We initially conducted 100 different experiments with the 10 meshes introduced in Section 2.1, aiming to answer the research question: What is the minimal number of independent camera views required to create an adequately complete digital twin of a microsurgical environment, using a narrow baseline camera setup? To this end, we qualitatively and quantitatively analyze the reconstructable subset of each mesh for different camera setups.

### 3.1. Benchmark

The use of 360 cameras in our setups is equivalent to distributing cameras in 1${}^{\circ }$ steps along the ring, hence placing a camera every 0.26 mm. This leads to the images of adjacent cameras overlapping almost completely, in turn meaning that any point that is visible to a camera on the ring is visible to at least one other camera. It is, therefore, safe to assume that 360 cameras provide the most complete possible reconstruction under the restrictions discussed in Section 2.2. Consequently, we use the surface that is reconstructable with 360 cameras as a benchmark. Since the meshes have different total areas, the results for each specific mesh were normalized to this maximum reconstructable area. This way, the results from different meshes can be directly compared to each other.

### 3.2. Experiment

We generated measurements with two, four, six, eight, 16, 32, 64, 128, 256, and 360 cameras for each of our 10 anatomic model meshes. The cameras were placed equidistantly on the ring. For example, two cameras would be arranged opposite of each other, four cameras every 90${}^{\circ }$, etc.

The camera setups always have a working distance of h=400mm and the cameras are always arranged on a ring with the diameter d=30mm. This diameter was chosen based on the maximum usable baseline in a ZEISS surgical microscope [25]. Together with the working distance, this led to a focal length of the simulated CMO lens of r≈400.28mm. This value lies well within the range of main objective focal lengths of surgical microscopes, which spans from 200 mm to 500 mm. The common focal point ($\vec{F}$) of all the cameras is the world coordinate origin (0,0,0), in a Cartesian coordinate system. The FOV of our simulated cameras is 36 mm× 24 mm in the focal plane. This FOV corresponds to that of an surgical microscope at the aforementioned working distance with 8× magnification.

Figure 5 illustrates a setup as described here, with four cameras and the 50 mm “circ artery” anatomic model.

Finally, assuming ideal circumstances for image acquisition and triangulation process, we consider a surface patch to be reconstructable if it is visible in two different cameras. Therefore, we chose a reconstructability threshold of T=2 for all experiments.

### 3.3. Qualitative Results

**Figure 8:**
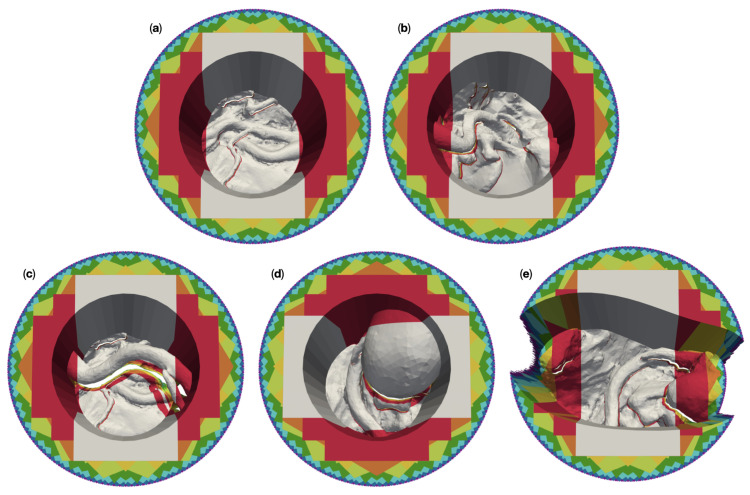
Overlay plots of the 3D reconstructable surfaces of the five different 50 mm-channel surgical site models for different camera numbers. The reconstruction with the largest area was placed in the background and the reconstruction with the smallest area in the foreground. The areas are color coded by number of cameras: Gray for two cameras, red for four cameras, orange for six, yellow for eight, lime for 16, green for 32, cyan for 64, blue for 128, purple for 256, and magenta for 360. (**a**) shows the reconstruction of “circ flat anatomy”. (**b**) does this for “circ angled anatomy”, (**c**) for “circ artery”, (**d**) for “circ overhang”, and (**e**) for the “elliptical channel” mesh.

Figure 8 shows the 3D-reconstructable surface of the 50 mm channel versions of the meshes (a) “circ flat anatomy”, (b) “circ angled anatomy”, (c) “circ artery”, (d) “circ overhang”, and (e) “elliptical channel”, for different numbers of cameras. For this purpose, we overlaid the individual reconstructable surfaces of the respective meshes. The reconstruction with the largest area was placed in the background and the reconstruction with the smallest area was placed in the foreground. The reconstructions were colored according to the number of cameras used. The gray area represents what can be reconstructed with two cameras. The additional surface area which is reconstructable by four cameras is shown in red, orange for six, yellow for eight, lime for 16, green for 32, cyan for 64, blue for 128, purple for 256, and magenta for 360. The top view is slightly tilted to show obscured regions which are not reconstructable.

For “circ flat anatomy”, the majority of the increase in reconstructable area with increasing camera number comprises sections of the operative channel walls. This can be seen in Figure 8a. This behavior still applies if the relevant anatomy is not located only at the bottom of the operative channel, and instead rises towards one side of the channel, as shown in Figure 8b.

**Figure 9:**
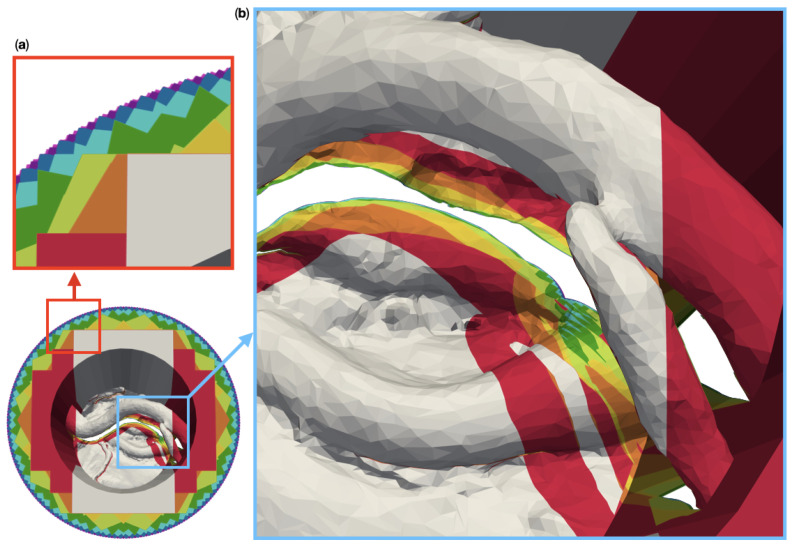
Magnified images of the 3D reconstructable surface of the model “circ artery”. The reconstruction with the largest area was placed in the background and the reconstruction with the smallest area in the foreground. (**a**) shows that the reconstructable area towards the outside of the top surface increases noticeably up to 128 cameras. After that, the increase is difficult to visually. (**b**) shows part of the model in which a vessel with a branch obscures the anatomy below. Large portions of this area are not reconstructable with two cameras (gray). In contrast to this, with four cameras, the red portions become available. The areas in different colors represents additional areas reconstructable with six (orange), eight (yellow), 16 (lime), 32 (green), 64 (cyan), 128 (blue), 256 (purple), and 360 (magenta) cameras.

This observation no longer applies in cases where an occluding structure is located high above the bottom of the channel, such as in “circ artery” (Figure 8c). In such cases, increasing the number of cameras does not only add portions of the channel wall to the reconstructable area, but also significant portions of the anatomical structures towards the bottom of the channel. This is shown in greater detail in Figure 9. The mesh “circ overhang” in Figure 8d exhibits a comparable effect.

A change in the operative channel geometry and cross section (here from circular to deformed elliptical) has no recognizable influence on the 3D reconstruction in the central area of the channel, as can be seen in Figure 8e. However, the outer regions (to the left and right of the image) require a far greater number of cameras for reconstruction in our “elliptical channel” model than with the circular channel models. Furthermore, the left and right sides of the channel walls are not reconstructable here, even with 360 cameras.

**Figure 10:**
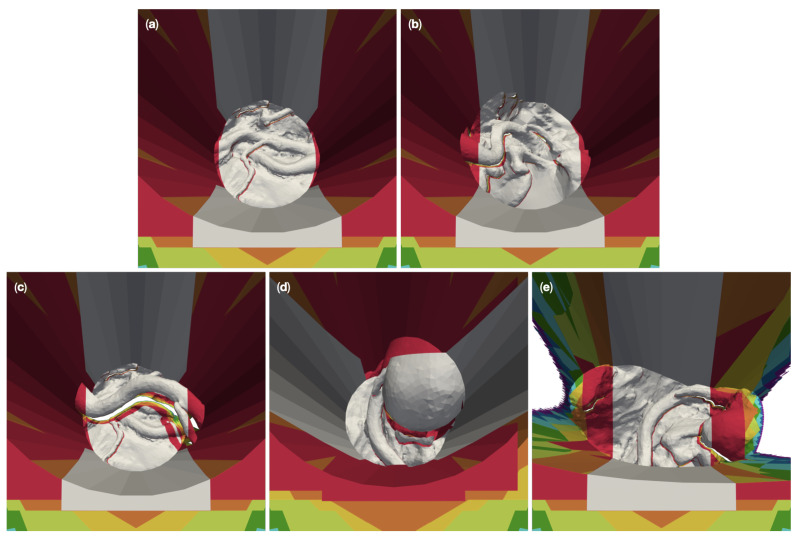
Overlay of zoomed plots of the 3D reconstructable surfaces of the five different 100 mm-channel surgical site models for different camera numbers. The reconstruction with the largest area was placed in the background and the reconstruction with the smallest area in the foreground. The areas are color coded by number of cameras: Gray for two cameras, red for four cameras, orange for six, yellow for eight, lime for 16, green for 32, cyan for 64, blue for 128, purple for 256, and magenta for 360. (**a**) shows the reconstruction of “circ flat anatomy”. (**b**) does this for “circ angled anatomy”, (**c**) for “circ artery”, (**d**) for “circ overhang”, and (**e**) for the “elliptical channel” mesh.

The results from our experiments with the 100 mm deep circular channels show qualitatively similar results to 50 mm models for the bottom part of the channel. This can be seen in Figure 10a–d. However, six cameras are required for the complete reconstruction of the deeper channel walls in these models, as opposed to the four cameras needed for in 50 mm channels.

In the case of the 100 mm deep elliptical model, like with the 50 mm version, no number of cameras could reconstruct the left and right portions of the channel walls (see Figure 10e). The number of cameras required to reconstruct the of the outer left and right portions of the anatomy in the channel also remains largely unchanged by channel depth.

### 3.4. Quantitative Results

Table 2 shows the reconstructable areas of the 50 mm channel models for different numbers of cameras. The area values for each mesh are normalized to the area of the respective mesh which is reconstructable with 360 cameras. This is always the maximum reconstructable area for any given mesh. Table 2 further lists the absolute and relative increases in reconstructable area from the next smaller number of cameras. The same information is provided in Table 3 for the 100 mm channel models.

In the following, we refer to the reconstructable area of a mesh as $A_{r}$. $A_{r}\left(n\right)$ denotes the area of a mesh that is reconstructable with *n* cameras. $\Delta A_{r}^{n\to m}$ signifies the reconstructable area that is added by increasing the number of cameras in the setup from *n* to *m*. $\Delta A_{r}$ alone refers to any change in reconstructable area of a given mesh.

#### 3.4.1. Results for 50 mm Channel Depth

As can be seen in Table 2, the $A_{r}$ of the different models with circular cross section are very similar for a given number of cameras. The elliptical channel model behaves quantitatively differently than the qualitative analysis has suggested.

In the case of the circular channel models, 55.01–57.03% of the maximum area ($A_{r}\left(360\right)$) can be reconstructed with two cameras. This fraction increases with the number of cameras, as observed in Section 3.4. Among all the camera number increments in the table, the step up from two to four cameras yields the greatest relative increase in reconstructable area. $\Delta A_{r}^{2\to 4}$ ranges from 53.93–63.41% for the circular channel models, which amounts to approximately twice the $\Delta A_{r}^{2\to 4}$ observed in the elliptical channel model. $\Delta A_{r}$ for the increments thereafter never exceeds 4.5% for the circular channel models, showing alternating behavior between six and 16 cameras. The elliptical channel model exhibits the same general behavior, albeit with greater values of $\Delta A_{r}$ than in the circular channel models. In addition, the alternating behavior of $\Delta A_{r}$ ends at 64 cameras here, instead of 16, as in the case of the circular channel models.

$A_{r}\left(32\right)$ is least 96.38% of $A_{r}\left(360\right)$ for the circular channel models. In the case of the elliptical model, twice the number of cameras are needed for the same result with $A_{r}\left(64\right)$ being 95.03% of $A_{r}\left(360\right)$.

#### 3.4.2. Results for 100 mm Channel Depth

The $A_{r}\left(2\right)$ of the 100 mm circular channel models are smaller than the $A_{r}\left(2\right)$ in their 50 mm counterparts by approximately 10 percentage points—decreasing from previously 55.01–57.03% to now 45.93–46.74%, as can be seen in Table 3.

$\Delta A_{r}^{2\to 4}$ is far greater in the 100 mm circular channels when compared to their 50 mm versions. Here, $\Delta A_{r}^{2\to 4}$ grows from 63.41% to 97.52%. Four cameras, therefore, fully double $A_{r}$ over two cameras, achieving $A_{r}\left(4\right)\approx 0.9\;\cdot \;A_{r}\left(360\right)$.

In the elliptical channel models, there is no growth in $\Delta A_{r}^{2\to 4}$ from 50 mm to 100 mm channel depth. Instead, $\Delta A_{r}^{2\to 4}$ shrinks from 29.9% in the 50 mm version to 21.65% in the 100 mm channel.

For the 100 mm circular channel meshes, the $\Delta A_{r}$ decrease steadily after $\Delta A_{r}^{2\to 4}$. This differs from the alternating behavior observed in the 50 mm models. The maximum $\Delta A_{r}$ above four cameras for all 100 mm circular channel models is 4.15%. For the increments beyond 64 cameras, the $\Delta A_{r}$ are all less than 1%. In these circular channel models, 95% of $A_{r}\left(360\right)$ can be achieved with only eight cameras, as opposed to the 16 cameras needed for the same result in the 50 mm channel models.

The 100 mm elliptical channel model is an exception yet again. In its case, the $\Delta A_{r}$ for the increments above four cameras range up to 19.29%, compared to a maximum of 4.15% with the circular channel models. Moreover, the $\Delta A_{r}$ here do not decrease uniformly, instead alternating as was the case with the 50 mm channel models.

### 3.5. Quantitative Results Excluding Mesh Top Surface

The images and observations in Section 3.3 show that much of the $\Delta A_{r}$ for increasing numbers of cameras occurred on the top surface. For $\Delta A_{r}^{4\to 6}$, and above, the majority of the addition in reconstructable area was located on the top surface. The higher the number of cameras, the more the respective $\Delta A_{r}$ is dominated by the top surface. The only exceptions to this rule are, again, the elliptical channel models.

The top surface is far from the focal point of the camera setup (Figure 5). We assume this focal point to also be the point interest of the surgeon. By extension, the region of highest interest surrounds this point. The top surface is, therefore, likely to be of less interest.

In consideration of the previous explanations, to facilitate later assessment of the practical value of the $\Delta A_{r}$ presented here, we chose to report our data a second time under exclusion of the top surface. The normalized results for the 50 mm deep channels with the excluded top surface are shown in Table 4. The results for the 100 mm deep channels are listed in Table 5.

#### 3.5.1. Results for 50 mm Channel Depth

A comparison of Table 2 and Table 4 shows that removing the top surface increases the $A_{r}\left(2\right)$ of the 50 mm deep circular channel meshes by approximately 7–9 percentage points. $A_{r}\left(2\right)$ of the 50 mm elliptical channel mesh increases by only approximately 2 percentage points. For the circular channel models, $\Delta A_{r}^{2\to 4}$ is decreased by approximately 5 percentage points by exclusion of the top surface, while it remains virtually unchanged for the elliptical channel mesh (+0.09 percentage points).

Comparing the new results to the simulations including the top surface, there is a clear shift towards lower camera numbers required to achieve 99% of $A_{r}\left(360\right)$ in all circular channel models. While it took 128 cameras to reach this number before removal of the top surface, it now requires four (“circ flat anatomy”), eight (“circ angled anatomy” & “circ overhang”), and 16 cameras (“circ artery”), respectively. Again, the elliptical channel results diverge strongly from the circular channel results.

In contrast to the alternating behavior observed previously in the 50 mm experiments including the top surface, the $\Delta A_{r}$ now decreases continuously above 6 cameras after removal of the top surface. “Circ angled anatomy” constitutes an exception, since there, $\Delta A_{r}^{8\to 16}$, with 0.47 percentage points, lies above both its neighboring values. The mesh with the elliptical channel is another exception, here the changes in $\Delta A_{r}$ are non-continuous and greater than in the meshes with a circular channel.

#### 3.5.2. Results for 100 mm Channel Depth

Finally, the results for the mesh with a channel depth of 100 mm with excluded top surface are shown in Table 5. After removal of the top surface, $A_{r}\left(2\right)$ increases by approximately 3 percentage points over the previous results for all 100 mm circular channel models. For these models, $\Delta A_{r}^{2\to 4}$ is decreased by approximately 4 percentage points by exclusion of the top surface, $\Delta A_{r}^{2\to 4}$ for the 100 mm elliptical channel mesh decreases by 1.66 percentage points.

Like in the 50 mm models, removal of the top surface causes a clear shift towards lower camera numbers required to achieve 99% of $A_{r}\left(360\right)$ in all circular channel models. While this took 64 cameras before removal of the top surface, it now requires six (“circ flat anatomy”, “circ angled anatomy”, & “circ overhang”) and 16 cameras (“circ artery”), respectively. Again, the elliptical channel results diverge strongly from the circular channel results.

### 3.6. Quantitative Results for Elliptical Channel Models Using a Larger Camera FOV

The elliptical channel model results invariably did not match the results from the circular channels models, both in trends and actual numbers. This was unexpected and raised the question as to the exact cause.

**Figure 11:**
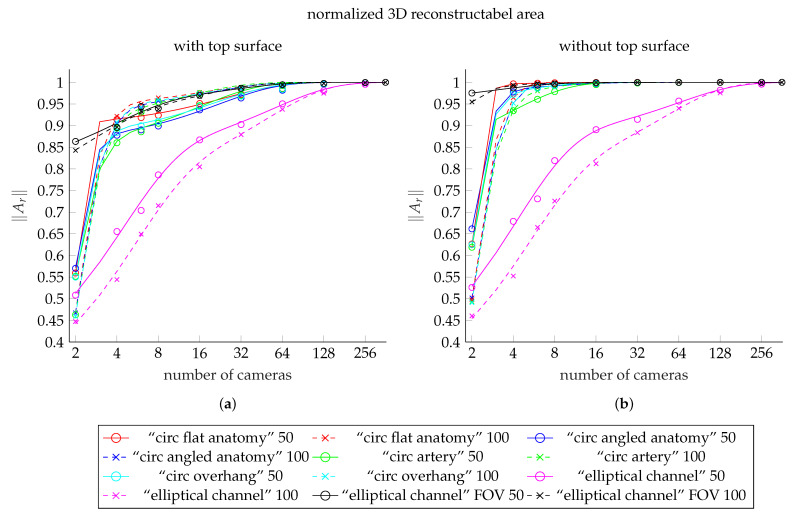
Comparison plot showing the fit functions to the reconstructable area values for each of our meshes. (**a**) shows the fit functions including the top surface, (**b**) shows the fits to data excluding the top surface. The curves for the meshes with 50 mm deep channel are solid lines. The dashed lines shows the curves of the meshes with 100 mm deep channel. The colors red, blue, green, and cyan represent the meshes “circ flat anatomy”, “circ angled anatomy”, “circ artery”, and “circ overhang”, in this order. Magenta belongs to the elliptical channel models, using the smaller FOV. The black curves belong to elliptical channel fits with larger FOV. The scattered markers represent the measured area values.

The results from the circular channel models all exhibit a very similar exponential dependency on the camera number, see Figure 11. Differences are limited mostly to the values of $A_{r}$. Channel depth and anatomic features could, therefore, be ruled out as the cause for the deviant behavior of the elliptical channel models. One of the remaining possible causes was the different channel cross-section.

However, we chose to further investigate the hypothesis that the discrepancies were caused by the camera FOV being too small for the elliptical channel models. This seemed the more likely cause, since the reconstructable region using four cameras covered only the central portion of the elliptical channel and its base. Yet the same number of cameras was sufficient to reconstruct the vast majority of the circular channel walls. This becomes clear upon comparison of subfigure (e) with the subfigures (a–d) in the respective plots for 50 mm and 100 mm channel depth in Figure 8 and Figure 10.

Our hypothesis was further supported by the much slower decrease of $\Delta A_{r}$ for the elliptical channel models, when compared to the circular channel models.

Finally, excluding the top surface drastically decreased the difference between $A_{r}\left(360\right)$ and $A_{r}\left(2\right)$ for circular channel models, in effect reducing the number of cameras needed to attain $A_{r}=0.99\;\cdot \;A_{r}\left(360\right)$ by an order of magnitude. This was not the case for the elliptical channels, further pointing towards the FOV as the cause.

All observations used in our reasoning here were discussed in the previous sections and can be validated by examining Table 2, Table 3, Table 4 and Table 5.

To investigate our hypothesis, we performed an additional 40 experiments with the elliptical channel models. In these experiments, we used the exact settings described in Section 3.2 with exception of the camera FOV, which was increased to 60 mm × 60 mm.

#### Results for Elliptical Channel Models using Cameras with Larger FOV

Table 6 shows the results of our additional experiments on the 50 mm and 100 mm elliptical channel models, respectively, including and excluding the top surface. All values were normalized to $A_{r}\left(360\right)$ as with the results of the other tests before. Comparing the results in Table 6 with those in Table 2 and Table 3, the most prominent changes with the larger FOV over the experiments with smaller FOV are the pronounced increases of $A_{r}\left(2\right)$ in all cases. Where $A_{r}\left(2\right)$ previously ranged from approximately 45–53% of $A_{r}\left(360\right)$ with the smaller FOV, $A_{r}\left(2\right)$ increased to between 84% and 98% $A_{r}\left(360\right)$ after increasing the FOV size. This means that two cameras are now sufficient to reconstruct a vast majority of the maximum possible reconstructable area. Consequently, all values of $\Delta A_{r}$ for every elliptical channel model also become much smaller after increasing the FOV.

The $\Delta A_{r}$ mostly decrease continuously after $\Delta A_{r}^{4\to 6}$ now, with $\Delta A_{r}^{6\to 8}$ being the only exception, as it is invariably smaller than $\Delta A_{r}^{4\to 6}$. $\Delta A_{r}^{6\to 8}$ for the 50 mm elliptical channel model including the top surface is even more of an outlier. It is the only case of negative $\Delta A_{r}$, i.e., a decrease in reconstructable area despite the increase in number of cameras, which we found in any of our experiments.

In general, the number of cameras needed to achieve 99% of $A_{r}\left(360\right)$ has been reduced greatly for all elliptical channel models by increasing the size of the FOV. Where 256 cameras were needed for this purpose in all previous cases, this number has dropped to 64 in the two experiments with the top surface. In the experiments excluding the top surface, six cameras were needed with the 50 mm channel, while the 100 mm channel required only four.

### 3.7. General Observations

The reconstructable area $A_{r}$ follows negative n-phase exponential decay functions in all cases ($n\in \left\{2,3\right\}$). This was found after fitting the functions shown in Figure 11 to the data. In our result, we could therefore not observe an obvious dependence of the reconstructable area on either the exact anatomy in the channel, nor on the channel geometry itself. This is, however, only true as long as the entire channel lies within the combined FOV of the camera setup.

Figure 11a shows the fits to the results from our models including the top surface. These values are listed in Table 2, Table 3, and Table 6. The fits to the corresponding results after exclusion of the top surface (Table 4, Table 5 and Table 6) are shown in Figure 11b.

The curves of the fit functions for 50 mm channel models are shown as solid lines. Dashed lines indicate curves of functions fitted to the results from models with 100 mm channels. The colors red, blue, green, and cyan represent the meshes “circ flat anatomy”, “circ angled anatomy”, “circ artery”, and “circ overhang”, in this order. The curves shown in magenta show the fit functions for meshes with elliptical channel, using the smaller FOV. The black curves belong to elliptical channel fits with larger FOV. The scattered markers show the measured values.

The absolute values for the experiment are shown in the Appendix A. Table A1 shows the results for meshes including the top surface, Table A2 for the meshes after excluding the top surface and Table A3 for meshes with elliptical channel.

## 4. Discussion and Conclusions

Our simulation framework is able to simulate the 3D reconstruction of representative models of microsurgical sites with different numbers of narrow-baseline cameras. The results from our simulated reconstructions, reported in Section 3, confirm our hypothesis: At a finite number of cameras, the 3D reconstructable area of the digital twin reaches a point where a further increase in camera views yields only a negligible increase in the reconstructable area. During the evaluation of our results, we found unexpected oscillating behavior in the increase of the 3D reconstructable area with increasing number of cameras. This was caused by the dependency of the reconstructable region of the top surface on the changing angular separation between the cameras at different camera numbers—and subsequently on the differing patterns in which their FOVs overlapped. The increases in area on the top surface also far outweighed the relatively small gains around the anatomical structures in the channel, diluting the results and making them difficult to interpret. All the while, the top surface is not relevant to the practical use of surgical microscopes: In surgical procedures, this region is only of immediate interest during the initial opening of the channel; a step done entirely without the aid of a surgical microscope in practice. Afterwards, the region is no longer of interest and is covered by surgical drapes. It is, therefore, essentially invisible to the microscope user during the operation and can, hence, be treated as of no interest for the rendering of co-observer views. Consequently, we based our conclusions only on the results of our experiments excluding the top surface.

It is also important to reiterate that the values given for the reconstructable areas in our results constitute theoretical maxima. We chose not to explicitly model any 3D reconstruction method, instead assuming any point to be correctly 3D reconstructable if is visible to at least cameras (see Section 2.2). The influences of aberrations, depth of field, lighting effects, and 3D reconstruction, therefore, still await investigation in future. We expect these error sources to strongly impact the visual quality and depth accuracy of the reconstruction. However, aberrations and the reconstruction method itself should not significantly change the reconstructable subset of the mesh itself. Only non-uniform lighting or insufficient depth of field should be able to entirely prevent the reconstruction of significant surface regions.

The relationships between the reconstructable area and the number of cameras in each of our experiments could uniformly be described by negative n-phase exponential decay functions. We did not find any evidence that this relationship depended either on the exact anatomy located in the channel, or on the channel geometry itself—provided that the channel and its contents lie entirely within the FOV of the camera setup.

We found out that two cameras, as currently used in the ZEISS KINEVO^®^, are too few, since they allow the reconstruction of only approximately 50% A(360) for each of the models. As explained in Section 3, 360 cameras are able to reconstruct every region of the input model that is visible from any viewing angle available within the simulated microscope.

Four cameras, as currently used in *Munich Surgical Imaging*’s ARRISCOPE, provide 95% of the performance of 360 cameras, while eight cameras reach 99%. Doubling the number of cameras from eight to 16 only adds performance in the range of $10^{-2}$% of that of 360 cameras. The gains thereafter become even more minuscule.

**Figure 12:**
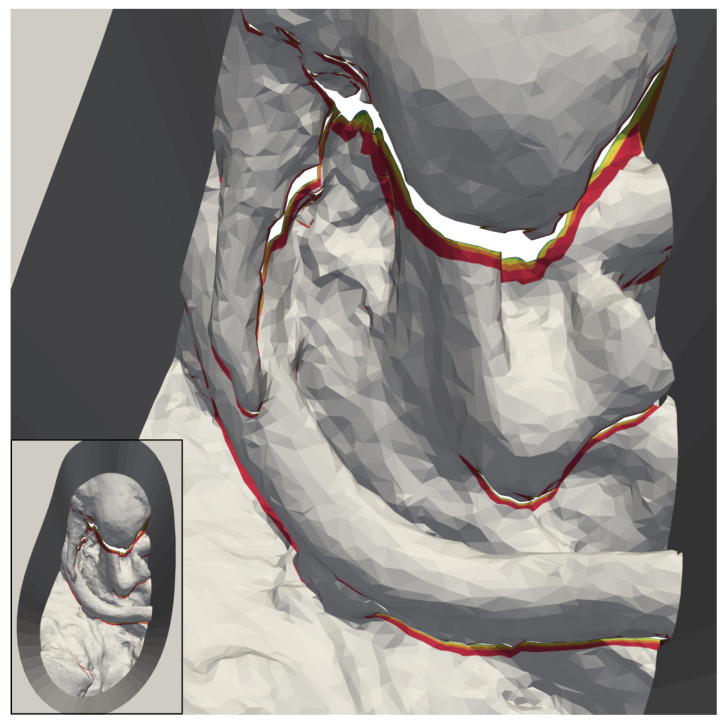
Reconstructable subset of the 50 mm deep version of our “elliptical channel” model, using cameras with increased FOV size. The regions shown in grey are reconstructable with only two cameras. The area added by four cameras is red. In addition, shown are additional areas reconstructable with six (orange), eight (yellow), 16 (lime), and 32 (green) cameras.

The colored areas in Figure 12 help illustrate this, using the 50 mm “elliptical channel” model as an example. Above a number of four cameras (red region), additional cameras only add small increments to the fringes of the reconstructable surface beneath occluding features. The yellow stripes, added by eight cameras, can still be distinguished without zooming in some areas, while the regions added by higher numbers of cameras can barely be seen.

The only case in our results, where one might argue that 16 cameras add practical value, is shown in Figure 9. The artery high above the rest of the anatomy in the channel occludes large regions below in many camera views. It, therefore, takes an unusually high number of cameras to make the areas underneath reconstructable. Even the regions added by 16 cameras (lime) are easily visible in the image in this case. The 3D reconstruction of surgical sites in our concept of a fully digital microscope, as outlined in Section 1, seems to be vulnerable to large vertical distances between anatomical features in operative channels. The extent of this vulnerability, as well as its consequences for the number of cameras needed for our use-case, require further exploration.

Based on the observed behavior a general answer can be given to the research question, which was: What is the minimal number of independent camera views are required to create an adequately complete digital twin of a microsurgical site, using a narrow baseline common main objective camera setup?

The number of cameras required for a 3D reconstruction is limited and largely the same for all channel models. Four cameras can yield a reconstruction sufficient for the generation of additional virtual views of the surgical field. Eight cameras are close to optimal and might be attainable if the additional cost and complexity for the 3D reconstruction over over four cameras can be managed. Any number of cameras above eight has little practical use, except perhaps if anatomical features are expected to reach far into the operative channel high above other anatomy of interest.

Our results further indicate that the suggested equidistant arrangement of cameras on the equivalent of a maximum baseline ring has the potential to yield reconstructions that are very close to optimal in a real surgical microscope under the given restrictions on viewing angles. Beder et al. [28] previously found that images taken by cameras 90${}^{\circ }$ apart yielded the highest depth accuracy for the 3D reconstruction of points. This gives us reason to believe that our suggested camera arrangement also has the potential for high depth accuracy. To exploit this potential, the arrangement might have to be logically split into camera pairs 90${}^{\circ }$ apart, using these for reconstruction instead of other possible camera combinations from this setup. Which camera view pairs to choose for reconstruction is, therefore, also a possible question for future analysis.

We must stress that the numbers of independent camera views stated above constitute only a lower limit for reconstruction of an adequately complete digital twin under optimal circumstances. Furthermore, by using only deep-channel models, we have have limited ourselves to the worst case scenario for microscope-borne 3D reconstruction. Sites with shallower channels or sites on the surface of the body (e.g., in ophthalmology) might require less cameras for reconstruction.

Any number of cameras named here is not guaranteed to yield a digital twin of the same quality in reality as in our simulations. In practice, 3D reconstruction will suffer from problems, such as insufficient feature points in the images for feature matching between camera views. A physical test setup for 3D reconstruction with a CMO microscope must be used to verify our findings in a future study.

In summary, we introduced a simulation framework that finds the most extensive subset of a model that could theoretically be 3D reconstructed with a given camera constellation, assuming ideal optics and disregarding any losses or errors introduced by the reconstruction method itself. We also introduced in silico models of typical deep-channel surgical sites, as well as a simplified in silico model of a CMO-pattern surgical microscope. Using the models and the simulation, we showed that a minimum of eight independent cameras views is preferable for 3D reconstruction of a deep channel surgical site if the complexity of implementation is manageable. Otherwise, a minimum of four independent views is sufficient for an adequately complete reconstruction. This study has laid the foundation for the digitization of surgical sites entirely in 3D. We have answered one of the key questions for the development of a prototype surgical 3D visualization system which offers currently unattainable features. A system that adapts to the user, instead of the user having to adapt to it. It will enable any number of independent assistants’ during operations, e.g., for those present in the operating room, as well as for remote training or expert assistance. It will also make 3D information fusion possible in surgical visualization systems.

## 5. Outlook

The presented models cover various geometries, but validation requires a sufficient in vivo data set. Validation on different in vivo geometries and channel depths could help to clarify whether the suggested annular camera arrangement is equally suitable in vivo.

Occlusion caused by the depth of the channels, as well as occluding structures inside the channels, are likely to blame for the high number of cameras required for reconstruction in our findings. In contrast, it would therefore be interesting to investigate the 3D reconstructability of a flat and wide-open site, such as the anterior chamber of the human eye. Given the absence of occluders, it might even be possible to achieve a complete 3D reconstruction of such a site with as little as four cameras, a small step up from the two cameras currently used in commercially available devices, such as the ZEISS ARTEVO 800^®^ [29].

As we mentioned earlier in the introduction, we believe that a 3D digital-twin based visualization approach can also be used in endoscopic and laparoscopic surgery –– promising information fusion, as well as free choice of view point for any number of observers. However, the feasibility of 3D reconstruction within the confines of hollow organs and laparoscopic approaches would have to analyzed separately. These use-cases are subject to very different constraints to camera numbers, stereo baseline, etc., than those applicable to the microsurgical use-case we chose to examine.

Our current simulation finds the subset of a surgical site model that is theoretically accessible to reconstruction with a given camera setup. It cannot simulate the actual reconstruction and, therefore, cannot convey insights as to the visual quality and depth accuracy that is to be expected from a setup under test. This will require a new simulation. The pinhole camera models should be replaced with a rendering pipeline for simulated acquisition of raster images. This pipeline should use a realistic camera model, allowing for lens aberrations and depth of field. The current projection matrix should, therefore, be replaced by a more sophisticated projection matrix with additional intrinsic parameters, e.g., of the CMO lens. Furthermore, the simple continuous-space threshold-based 3D reconstructability decision on polygons would have to be replaced with a discrete-space photogrammetric 3D reconstruction algorithm. Finally, a realistic lighting model should be incorporated into the new simulation, thus taking the effects of lighting on the 3D reconstruction into account.

Once improved in silico studies are done, results from the in silico model can be used to implement an experimental prototype setup. The prototype setup and ex vivo experiments can also be used to investigate how inaccuracies, e.g., in feature matching, affect the accuracy of 3D reconstruction. At least, in vivo studies are needed to finally state the accuracy in clinical use. 

## Figures and Tables

**Table 1:** Operation channel diameters for groups of lesions that are of particular interest in microsurgery. We used these values as a basis for the design of our operation channel models. All values are given in millimeters. Data provided by Carl ZEISS Meditec AG.

Organ	Operation Type	Diameter Range	Average Diameter	Depth Range
**Brain**	Glioblastoma resection	40–70	55	40–90
	Interventions in the cerebellopontine angle	20–40	30	60–100
**Spine**	Intervertebral disc surgery	20–30	25	50–100

**Table 2:** Reconstructable areas ($A_{r}$) of the 50 mm channel models for different numbers of cameras, including the top surface. The area values for each mesh are normalized to the area of the respective mesh which is reconstructable with 360 cameras. $\Delta A_{r}$ denotes the increase in reconstructable area from the next smaller number of cameras.

Number of	“Circ Flat Anatomy”			“Circ Angled Anatomy”			“Circ Artery”			“Circ Overhang”			“Elliptical Channel”		
Cameras	$∥A_{r}∥$	$\Delta A_{r}$		$∥A_{r}∥$	$\Delta A_{r}$		$∥A_{r}∥$	$\Delta A_{r}$		$∥A_{r}∥$	$\Delta A_{r}$		$∥A_{r}∥$	$\Delta A_{r}$	
2	0.5595	0		0.5703	0		0.5519	0		0.5501	0		0.5084	0	
4	0.9142	0.3548	(+63.41%)	0.8778	0.3076	(+53.93%)	0.8604	0.3085	(+55.9%)	0.8844	0.3343	(+60.77%)	0.6554	0.1469	(+28.9%)
6	0.9185	0.0043	(+0.47%)	0.8902	0.0124	(+1.41%)	0.8858	0.0253	(+2.94%)	0.8995	0.0151	(+1.71%)	0.704	0.0487	(+7.43%)
8	0.9239	0.0054	(+0.59%)	0.8990	0.0088	(+0.99%)	0.9054	0.0197	(+2.22%)	0.9098	0.0102	(+1.14%)	0.7861	0.0821	(+11.66%)
16	0.9511	0.0272	(+2.94%)	0.9363	0.0373	(+4.15%)	0.9461	0.0406	(+4.49%)	0.9437	0.034	(+3.73%)	0.867	0.0809	(+10.29%)
32	0.9719	0.0208	(+2.19%)	0.9638	0.0275	(+2.94%)	0.9701	0.0241	(+2.54%)	0.9681	0.0244	(+2.58%)	0.9025	0.0355	(+4.09%)
64	0.9857	0.0138	(+1.42%)	0.9817	0.0179	(+1.85%)	0.9851	0.0149	(+1.54%)	0.9838	0.0157	(+1.62%)	0.9503	0.0478	(+5.3%)
128	0.9963	0.0106	(+1.08%)	0.9952	0.0135	(+1.38%)	0.9961	0.011	(+1.12%)	0.9958	0.012	(+1.22%)	0.9807	0.0304	(+3.2%)
256	0.999	0.0027	(+0.27%)	0.9988	0.0035	(+0.35%)	0.999	0.0029	(+0.29%)	0.9989	0.0031	(+0.31%)	0.9955	0.0148	(+1.51%)
360	1	0.001	(+0.1%)	1	0.0012	(+0.12%)	1	0.001	(+0.1%)	1	0.0011	(+0.11%)	1	0.0045	(+0.46%)

**Table 3:** Reconstructable areas ($A_{r}$) of the 100 mm channel models for different numbers of cameras, including the top surface. The area values for each mesh are normalized to the area of the respective mesh which is reconstructable with 360 cameras. $\Delta A_{r}$ denotes the increase in reconstructable area from the next smaller number of cameras.

Number of	“Circ Flat Anatomy”			“Circ Angled Anatomy”			“Circ Artery”			“Circ Overhang”			“Elliptical Channel”		
Cameras	$∥A_{r}∥$	$\Delta A_{r}$		$∥A_{r}∥$	$\Delta A_{r}$		$∥A_{r}∥$	$\Delta A_{r}$		$∥A_{r}∥$	$\Delta A_{r}$		$∥A_{r}∥$	$\Delta A_{r}$	
2	0.4673	0		0.4674	0		0.4629	0		0.4593	0	0	0.4474	0	0
4	0.9214	0.4541	(+97.19%)	0.9044	0.437	(+93.49%)	0.8942	0.4314	(+93.19%)	0.9072	0.4479	(+97.52%)	0.5443	0.0969	(+21.65%)
6	0.9494	0.028	(+3.04%)	0.9396	0.0352	(+3.89%)	0.9313	0.0371	(+4.15%)	0.9428	0.0356	(+3.92%)	0.6493	0.105	(+19.29%)
8	0.9641	0.0147	(+1.55%)	0.9571	0.0175	(+1.86%)	0.9547	0.0234	(+2.51%)	0.9595	0.0168	(+1.78%)	0.7153	0.066	(+10.16%)
16	0.9767	0.0126	(+1.31%)	0.9728	0.0156	(+1.64%)	0.9741	0.0195	(+2.04%)	0.9746	0.015	(+1.57%)	0.8049	0.0896	(+12.52%)
32	0.986	0.0093	(+0.95%)	0.9839	0.0111	(+1.14%)	0.9851	0.011	(+1.13%)	0.9849	0.0104	(+1.07%)	0.8792	0.0743	(+9.23%)
64	0.9929	0.0069	(+0.7%)	0.9919	0.0081	(+0.82%)	0.9927	0.0075	(+0.77%)	0.9925	0.0075	(+0.76%)	0.9375	0.0583	(+6.63%)
128	0.9976	0.0047	(+0.47%)	0.9973	0.0053	(+0.54%)	0.9975	0.0048	(+0.49%)	0.9975	0.005	(+0.5%)	0.9749	0.0374	(+3.99%)
256	0.9993	0.0017	(+0.17%)	0.9992	0.0019	(+0.19%)	0.9993	0.0018	(+0.18%)	0.9993	0.0018	(+0.18%)	0.9944	0.0195	(+2%)
360	1	0.0007	(+0.07%)	1	0.0008	(+0.08%)	1	0.0007	(+0.07%)	1	0.0007	(+0.07%)	1	0.0056	(+0.57%)

**Table 4:** Reconstructable areas ($A_{r}$) of the 50 mm channel models for different numbers of cameras, excluding the top surface. The area values for each mesh are normalized to the area of the respective mesh which is reconstructable with 360 cameras. $\Delta A_{r}$ denotes the increase in reconstructable area from the previous smaller number of cameras.

Number of	“Circ Flat Anatomy”			“Circ Angled Anatomy”			“Circ Artery”			“Circ Overhang”			“Elliptical Channel”		
Cameras	$∥A_{r}∥$	$\Delta A_{r}$		$∥A_{r}∥$	$\Delta A_{r}$		$∥A_{r}∥$	$\Delta A_{r}$		$∥A_{r}∥$	$\Delta A_{r}$		$∥A_{r}∥$	$\Delta A_{r}$	
2	0.6264	0		0.6615	0		0.619	0		0.6249	0		0.5263	0	
4	0.9965	0.37	(+59.07%)	0.9776	0.3161	(+47.79%)	0.9343	0.3153	(+50.94%)	0.9723	0.3474	(+55.6%)	0.6788	0.1525	(+28.99%)
6	0.9979	0.0014	(+0.15%)	0.9883	0.0107	(+1.09%)	0.9608	0.0265	(+2.83%)	0.9865	0.0142	(+1.46%)	0.7308	0.052	(+7.66%)
8	0.9989	0.001	(+0.1%)	0.9921	0.0038	(+0.39%)	0.9786	0.0178	(+1.86%)	0.9927	0.0062	(+0.63%)	0.8191	0.0883	(+12.09%)
16	0.9996	0.0007	(+0.07%)	0.9968	0.0047	(+0.47%)	0.9947	0.0161	(+1.64%)	0.9977	0.005	(+0.5%)	0.8906	0.0715	(+8.73%)
32	0.9999	0.0003	(+0.03%)	0.9989	0.002	(+0.21%)	0.9984	0.0037	(+0.37%)	0.9994	0.0016	(+0.16%)	0.9144	0.0238	(+2.67%)
64	0.9999	0.0001	(+0.01%)	0.9996	0.0007	(+0.07%)	0.9995	0.0011	(+0.11%)	0.9998	0.0004	(+0.04%)	0.9567	0.0423	(+4.62%)
128	1	0	(+0%)	0.9998	0.0003	(+0.03%)	0.9998	0.0003	(+0.03%)	0.9999	0.0001	(+0.01%)	0.9817	0.025	(+2.61%)
256	1	0	(+0%)	1	0.0001	(+0.01%)	1	0.0002	(+0.02%)	1	0.0001	(+0.01%)	0.9958	0.0141	(+1.44%)
360	1	0	(+0%)	1	0	(+0%)	1	0	(+0%)	1	0	(+0%)	1	0.0042	(+0.42%)

**Table 5:** Reconstructable areas ($A_{r}$) of the 100 mm channel models for different numbers of cameras, excluding the top surface. The area values for each mesh are normalized to the area of the respective mesh which is reconstructable with 360 cameras. $\Delta A_{r}$ denotes the increase in reconstructable area from the next smaller number of cameras.

Number of	“Circ Flat Anatomy”			“Circ Angled Anatomy”			“Circ Artery”			“Circ Overhang”			“Elliptical Channel”		
Cameras	$∥A_{r}∥$	$\Delta A_{r}$		$∥A_{r}∥$	$\Delta A_{r}$		$∥A_{r}∥$	$\Delta A_{r}$		$∥A_{r}∥$	$\Delta A_{r}$		$∥A_{r}∥$	$\Delta A_{r}$	
2	0.4978	0		0.502	0		0.4934	0		0.4911	0		0.4604	0	
4	0.9645	0.4668	(+93.77%)	0.9519	0.4499	(+89.62%)	0.9359	0.4425	(+89.67%)	0.9519	0.4608	(+93.85%)	0.5525	0.092	(+19.99%)
6	0.999	0.0344	(+3.57%)	0.9949	0.043	(+4.52%)	0.9801	0.0443	(+4.73%)	0.9949	0.043	(+4.51%)	0.6649	0.1125	(+20.36%)
8	0.9995	0.0005	(+0.05%)	0.9966	0.0017	(+0.17%)	0.9898	0.0096	(+0.98%)	0.9967	0.0019	(+0.19%)	0.7257	0.0608	(+9.14%)
16	0.9999	0.0004	(+0.04%)	0.9986	0.002	(+0.2%)	0.9974	0.0076	(+0.77%)	0.9989	0.0022	(+0.22%)	0.8121	0.0864	(+11.9%)
32	1	0.0001	(+0.01%)	0.9995	0.0009	(+0.09%)	0.9992	0.0018	(+0.18%)	0.9997	0.0007	(+0.07%)	0.8838	0.0717	(+8.83%)
64	1	0	(+0%)	0.9998	0.0003	(+0.03%)	0.9998	0.0006	(+0.06%)	0.9999	0.0002	(+0.02%)	0.9398	0.0561	(+6.34%)
128	1	0	(+0%)	0.9999	0.0001	(+0.01%)	0.9999	0.0001	(+0.01%)	1	0.0001	(+0.01%)	0.9755	0.0357	(+3.79%)
256	1	0	(+0%)	1	0	(+0%)	1	0.0001	(+0.01%)	1	0	(+0%)	0.9946	0.0191	(+1.96%)
360	1	0	(+0%)	1	0	(+0%)	1	0	(+0%)	1	0	(+0%)	1	0.0054	(+0.54%)

**Table 6:** Reconstructable areas ($A_{r}$) of the “elliptical channel” models for different numbers of cameras using an larger FOV (60 mm × 60 mm). The area values for each mesh are normalized to the area of the respective mesh which is reconstructable with 360 cameras. $\Delta A_{r}$ denotes the increase in reconstructable area from the next smaller number of cameras.

Number of Cameras	Elliptical Channel with a Larger FOV											
	Including Top Surface						Excluding Top Surface					
	50 mm Deep Channels			100 mm Deep Channels			50 mm Deep Channels			100 mm Deep Channels		
	$∥A_{r}∥$	$\Delta A_{r}$		$∥A_{r}∥$	$\Delta A_{r}$		$∥A_{r}∥$	$\Delta A_{r}$		$∥A_{r}∥$	$\Delta A_{r}$	
2	0.8634	0		0.8427	0		0.9752	0		0.9547	0	
4	0.8959	0.0324	(+3.76%)	0.8966	0.0538	(+6.39%)	0.987	0.0118	(+1.21%)	0.9932	0.0385	(+4.03%)
6	0.944	0.0481	(+5.37%)	0.9334	0.0369	(+4.11%)	0.9933	0.0062	(+0.63%)	0.9965	0.0033	(+0.33%)
8	0.94	−0.004	(−0.42%)	0.9375	0.0041	(+0.44%)	0.996	0.0027	(+0.28%)	0.9979	0.0014	(+0.14%)
16	0.9699	0.0299	(+3.18%)	0.9675	0.03	(+3.2%)	0.999	0.003	(+0.3%)	0.9995	0.0016	(+0.16%)
32	0.9862	0.0163	(+1.68%)	0.9884	0.0209	(+2.16%)	0.9996	0.0006	(+0.06%)	0.9998	0.0003	(+0.03%)
64	0.9944	0.0082	(+0.83%)	0.9937	0.0053	(+0.54%)	0.9998	0.0002	(+0.02%)	0.9999	0.0001	(+0.01%)
128	0.9975	0.0031	(+0.31%)	0.9977	0.004	(+0.4%)	0.9999	0.0001	(+0.01%)	1	0	(+0%)
256	0.9995	0.002	(+0.2%)	0.9992	0.0015	(+0.15%)	1	0	(+0%)	1	0	(+0%)
360	1	0.0005	(+0.05%)	1	0.0008	(+0.080%)	1	0	(+0%)	1	0	(+0%)

## Data Availability

The MATLAB code of the framework is online available under GNU General Public License Version 3 together with 4 exemplary meshes at doi:10.5281/zenodo.4727697.

## Abbreviations

The following abbreviations are used in this manuscript:

MDPI	Multidisciplinary Digital Publishing Institute
FOV	Field of view
CMO	common main objective
STL	stereolithography
ROI	region of interest
AR	augmented reality
VR	virtual reality
3D	three-dimensional
$A_{r}$	reconstructable areas

## Appendix A. Absolute Result Values

**Table A1:** Absolute values of the models including the mesh top surface.

Number of Cameras	50 mm Deep Channel					100 mm Deep Channel				
	Circ Angled Anatomy	Circ Artery	Circ Flat Anatomy	Circ Overhang	Elliptical Channel	Circ Angled Anatomy	Circ Artery	Circ Flat Anatomy	Circ Overhang	Elliptical Channel
2	2180.53	2606.64	2693.70	2362.84	2654.16	3537.98	3890.15	3969.95	3692.46	3954.95
4	3356.55	4063.70	4401.76	3798.81	3421.09	6845.67	7515.49	7828.25	7293.31	4811.07
6	3404.02	4183.30	4422.30	3863.85	3675.29	7111.80	7827.31	8066.09	7579.15	5739.22
8	3437.72	4276.11	4448.32	3907.83	4103.85	7244.43	8023.60	8191.01	7713.91	6322.41
16	3580.24	4468.04	4579.21	4053.70	4526.04	7362.88	8187.21	8298.20	7834.79	7114.16
32	3685.43	4581.74	4679.38	4158.41	4711.22	7447.15	8279.58	8377.04	7918.24	7771.07
64	3753.73	4652.29	4745.77	4225.98	4960.89	7508.13	8342.94	8436.05	7978.77	8286.51
128	3805.53	4704.30	4796.90	4277.46	5119.40	7548.59	8383.69	8475.88	8018.96	8616.84
256	3819.01	4718.06	4810.08	4290.81	5196.54	7563.16	8398.45	8490.17	8033.40	8789.11
360	3823.74	4722.80	4814.70	4295.50	5220.23	7569.11	8404.50	8496.04	8039.31	8839.03

* all values are in mm${}^{2}$.

**Table A2:** Absolute values of the models excluding the mesh top surface.

Number of Cameras	50 mm Deep Channel					100 mm Deep Channel				
	Circ Angled Anatomy	Circ Artery	Circ Flat Anatomy	Circ Overhang	Elliptical Channel	Circ Angled Anatomy	Circ Artery	Circ Flat Anatomy	Circ Overhang	Elliptical Channel
2	2029.79	2455.90	2542.95	2212.10	2484.92	3508.06	3860.23	3940.03	3662.54	3902.41
4	2999.76	3706.91	4044.98	3442.03	3205.17	6652.06	7321.87	7634.64	7099.7	4682.31
6	3032.57	3811.85	4050.85	3492.40	3450.65	6952.80	7668.31	7907.09	7420.15	5635.57
8	3044.26	3882.64	4054.86	3514.36	3867.70	6964.56	7743.74	7911.15	7434.05	6150.52
16	3058.60	3946.40	4057.57	3532.06	4205.32	6978.78	7803.12	7914.10	7450.7	6882.52
32	3064.87	3961.18	4058.83	3537.86	4317.55	6985.00	7817.42	7914.88	7456.08	7490.37
64	3067.11	3965.67	4059.15	3539.37	4517.09	6987.20	7822.01	7915.12	7457.84	7965.51
128	3067.90	3966.68	4059.27	3539.83	4635.15	6987.91	7823.02	7915.21	7458.29	8267.71
256	3068.27	3967.33	4059.35	3540.08	4701.71	6988.23	7823.53	7915.25	7458.48	8429.70
360	3068.40	3967.46	4059.36	3540.15	4721.64	6988.33	7823.72	7915.26	7458.53	8475.36

* all values are in mm${}^{2}$.

**Table A3:** Absolute values of the “elliptical channel” models with a larger FOV.

Number of Cameras	Elliptical Channel with a Larger FOV			
	Including Top Surface		Excluding Top Surface	
	50 mm Deep Channel	100 mm Deep Channel	50 mm Deep Channel	100 mm Deep Channel
2	6871.89	11,019.57	5073.47	10,056.69
4	7130.12	11,723.53	5134.85	10,461.92
6	7513.14	12,205.45	5167.31	10,496.57
8	7481.50	12,258.89	5181.59	10,511.21
16	7719.42	12,651.23	5197.12	10,527.66
32	7849.21	12,924.11	5200.28	10,531.26
64	7914.27	12,993.35	5201.46	10,532.68
128	7938.87	13,045.03	5201.84	10,533.09
256	7954.70	13,065.00	5202.09	10,533.29
360	7958.74	13,075.75	5202.31	10,533.44

* all values are in mm${}^{2}$.
